# A Care Concept of Community Health Nursing Interventions for Adults With Chronic Health Conditions in an Urban Area: Protocol for a Randomized Controlled Field Trial (CoSta Study)

**DOI:** 10.2196/37965

**Published:** 2022-09-28

**Authors:** Annike Morgane Nock, Linda Iversen, Lukas Waidhas, Antonia Zapf, Caroline Seifert, Corinna Petersen-Ewert

**Affiliations:** 1 Department of Nursing and Management Hamburg University of Applied Sciences Hamburg Germany; 2 Institute of Medical Biometry and Epidemiology Medical Center Hamburg-Eppendorf University Hamburg Hamburg Germany

**Keywords:** community health nursing, chronic health conditions, randomized controlled trial, community-based program, health-related quality of life, nurse-led consultation, nursing, nurse, intervention, urban, protocol, Germany, adults

## Abstract

**Background:**

Implementing community health nursing programs is a new field of application in the primary health sector of Germany. Hence, there is limited evidence of effective community-based and nurse-led interventions with regard to the German health care system. International research findings are mostly not transferable. The Community Health Nursing in der Stadt (CoSta; ie, “Community Health Nursing in the City”) project is the first study that examines a community health nurse–led intervention for adults with chronic health conditions.

**Objective:**

This study protocol describes the design and methods of a randomized controlled field trial that will investigate if a community health nurse–based intervention has an impact on health-related quality of life in adults with chronic conditions.

**Methods:**

The study was designed as a randomized controlled trial that will be conducted under real-life conditions in the field. In a 4-month period, patients with at least 1 chronic *International Classification of Diseases, Tenth Revision*, diagnosis will be enrolled. Participants will be randomly allocated to an intervention group or a control group. The sample size was assumed based on an effect size of 0.50 with a significance level of .05, using a 2-sided (2-tailed), 2-sample unequal variance *t* test. The control group will be treated as usual. The intervention group will receive—in addition to the usual treatment—preventive home visits; consultations; and educative training, which will be offered by 2 community health nurses for up to 12 months. Both groups will be followed up at baseline, after 6 months, and after 12 months. The primary outcome measure is the mental component summary score from the 36-Item Short Form Health Survey after 12-months. Secondary patient outcomes will be included. The study received ethics approval from the Competence Health Center’s institutional review board at the University of Applied Sciences Hamburg (procedure number: 2020-14).

**Results:**

The CoSta project was funded by the Federal Ministry of Education and Research Germany (contract number: 13FH019SX8). In total, 187 participants were recruited at the beginning of August 2021. Further, 92 were excluded and 94 were randomized. Data collection will be conducted until the end of 2022.

**Conclusions:**

Our study will provide data with regard to the effectiveness of community nurse–led interventions that focus on the treatment of vulnerable adults with chronic health conditions in a community health center. In secondary analyses, the associations among influencing social factors (education, income, and employment) will be examined. We expect results that will help reduce the research-to-practice gap.

**Trial Registration:**

German Clinical Trials Register DRKS00026164; https://tinyurl.com/yckxc5ut

**International Registered Report Identifier (IRRID):**

DERR1-10.2196/37965

## Introduction

### Background

The implementation of advanced nursing professionals in primary care has become a topic of public debate in Germany lately. The COVID-19 pandemic has emphasized already existing gaps and health inequalities in primary care and community settings. During the last 2 years, different studies reported a disproportionate impact on disease burden and well-being in vulnerable target groups, as well as socioeconomic disparities resulting from the pandemic event [1,2]. Neighborhoods with high rates of poverty, precarious employment, and discrimination are more affected by COVID-19 itself and the economic, psychological, and social consequences resulting from the pandemic [3]. Additionally, for people with (multiple) chronic health conditions, a higher risk of experiencing these effects, as a result of the pandemic event, has been reported [4]. Regional data on reported COVID-19 cases in Germany show increased mortality rates in socially disadvantaged districts [5], references to COVID-19–related outcomes (eg, infection rates), and correlations with social indicators [6]. The latest evidence does not differ from those reported in previous pandemics [7], revealing existing health-related inequity in societies [8] and underlining the important roles of community and public health services and authorities. In Germany, this evidence has intensified the discourse on gaps in the health care system. It is not news that primary care is under threat, particularly as there is a lack of health services in certain rural and urban districts that exacerbates unequal access, especially among socially disadvantaged populations [9]. Moreover, it is well known that there is a gap in life expectancy (up to 10 years between high-income and low-income individuals) in Germany. These differences also exist for the majority of people with chronic health conditions. Such health inequalities are largely the result of social determinants of health [10]. Therefore, using nurse-centered approaches in the community could be a sustainable solution. According to successfully implemented international role models, the community health nursing (CHN) concept [11] has been discussed for the primary health sector in Germany [12,13]. Nurse professionals at the master's degree level provide evidence-based care and health promotion in various settings (eg, in community health centers [CHCs]), addressing vulnerable groups and their particular needs [14]. The initiative of the Robert Bosch Foundation (with the Agnes Karll Society and the German Professional Association of Nursing) to establish CHN and test master's degree programs in Germany [13], as well as a recently published legal requirement [15], has advanced this ongoing debate. In other countries, such as Canada, this nurse profile has been implemented in health services for several years [16]. However, international research findings are mostly not directly transferable to the German health care system. The legal framework for CHN's scope of action is still lacking [13], and funding for CHN and CHN’s anchoring in the laws on benefits must be reviewed [13]. Accordingly, CHN still needs to be developed in Germany, and more information and evidence are needed about the effectiveness of community-based nurse interventions and their impact on socially determined health inequalities. The CHN in der Stadt (CoSta; ie, “Community Health Nursing in the City”) project is the first project that evaluates CHN interventions for patients with chronic health conditions who live in a disadvantaged neighborhood (Hamburg-Veddel). The social disadvantages that many residents experience in this neighborhood manifest as high unemployment rates, low household incomes, and a high dependence on state transfer payments, when compared to other districts of the city [17]. Furthermore, health challenges are reflected in the neighborhood by both the higher prevalence of chronic conditions, such as hypertonus, and the low utilization of outpatient care [18].

The CoSta project was funded in 2020 by the German Federal Ministry of Education and Research. For the project, a community nurse–led care concept targeting chronic conditions has been developed. The research questions of the CoSta study aim at exploring how community health nurse interventions impact health-related quality of life, health literacy, coping, depressive symptoms, anxiety, social participation, and the utilization of health services among patients with chronic health conditions. Furthermore, we assume that social disadvantage will correlate with the impact on chronic health conditions and the use of health care services. In the secondary analyses, we will focus on the effects of education, employment, and income on health. To answer the research questions, a randomized controlled trial (RCT) will be conducted for 1 year. The CoSta project is being realized via a joint effort by the University of Applied Sciences Hamburg and Community Health Centre Policlinic Veddel. Statistical advice has been provided by the Institute of Medical Biometry and Epidemiology at the Medical Center Hamburg-Eppendorf. The purpose of this study protocol is to describe the RCT, which was designed to examine the effectiveness of a new community health nurse concept among adults with chronic health conditions. This study protocol follows the SPIRIT (Standard Protocol Items: Recommendations for Interventional Trials) guidelines.

### Objectives

The aim of the CoSta study is to explore the effects of a new nurse health care concept on patients with chronic health conditions (ie, effects on primary and secondary outcome measures) and compare them to the effects of usual treatment. The primary alternative hypothesis is that participants in the intervention group will experience greater improvements in the mental components of their health-related quality of life compared to those experienced by participants in the control group over a 12-month period. The following research questions will be addressed in secondary analyses:

- How do the outcomes of patients regarding health-related quality of life, depressive symptoms, anxiety, health literacy, coping, social participation, and the utilization of health care differ between groups (nurse-led intervention group and usual care group)?
- How should nurse-led consultations be designed?
- How can nurse-led health promotion interventions be implemented in the neighborhood?

## Methods

### Trial Design

The CoSta study was designed as a prospective, monocentric, 2-armed RCT that will evaluate the effectiveness of community nurse–led interventions for adults with chronic conditions from an urban area in Germany. A 1:1 allocation ratio for the intended number of participants in each of the two groups will be used. The primary and secondary outcomes that will be assessed in the study are guided by the principle that defines *health* as “a state of complete physical, mental and social well-being and not merely the absence of disease or infirmity,” as stated in the Constitution of the World Health Organization [19]. The methodological procedure is based on the methods of a Canadian study that evaluated an interprofessional, nurse-led program for promoting self-management in older adults with multiple chronic conditions [20]. A participant timeline recommended by the SPIRIT guidelines was generated to visualize the planned process of enrollment, interventions, and selected validated assessment instruments (Multimedia Appendix 1).

### Study Setting

The study will enroll adult patients with chronic health conditions who are treated in a CHC and will involve a multiprofessional team of general practitioners, clinical psychologists, social workers, midwives, and advanced nurses who provide health care in the community. The clinic is located in the Hamburg-Veddel district, which can be described as a socially disadvantaged urban neighborhood wherein about 5000 people live in a densely built housing estate surrounded by railroad tracks, highways, and a port and industrial area. The residents are comparatively young, and many are dependent on state transfer payments [17]. Chronic health conditions, such as diabetes mellitus, hypertension, heart failure, and lung diseases, are highly prevalent [18]. Further, the amount of primary care offerings are comparatively below the average. Interprofessional cooperation will play a great part in developing the program. The two interventionists have their own office, and both participate in interprofessional case conferences and supervision meetings.

### Eligibility Criteria

The criteria that were chosen will allow for the broad inclusion of current patients with chronic diagnoses from the CHC. Participants who fulfill the following criteria will be enrolled: (1) patients who provide informed consent, (2) patients aged ≥18 years (no limit), (3) patients who are registered as patients at the CHC, (4) patients with a diagnosis of at least 1 chronic condition, and (5) patients who live at home.

Patients who do not fit the criteria for the study, refuse to participate, or are experiencing a severe course of disease will be excluded. Furthermore, people living with dementia or an acute psychiatric diagnosis and patients living in foster care will be excluded from enrollment.

### Interventions

The framework of the CHN concept is based on the “critical-emancipatory concept of nursing action” [21]. The CoSta program is a 12-month, patient-driven, multicomponent intervention that was developed to empower patients with chronic health conditions in managing their illness. It consists of the following three different types of nurse-led interventions: (1) home visits offered once per month by the community health nurse, (2) weekly consultations offered by the community health nurse, and (3) monthly training sessions that are tailored to the course of a chronic disease and are hosted by specialist practitioners or the interventionists themselves. During the home visits and consultations, assessments, physical examinations, and care coordination and planning will be carried out by the community health nurse. During the weekly consultations, assessments, care coordination and care navigation (linking participants to other health care services) will be conducted by the community health nurse. During 4 additional topic-centered consultation hours (each will occur once per month), which will be dedicated to cardiovascular diseases, lung diseases, chronic pain, and diabetes mellitus, as well as specific disease counseling, will be offered. Professional translators will be brought in for the interventions, as needed.

Patients who are randomly assigned to the intervention group will be offered all of the aforementioned interventions in addition to the usual treatment that they receive in the CHC. The interventionists will not deliver any health services to participants who are randomly assigned to the control group. These participants will be offered treatment as usual. Because the CHN concept involves patient-tailored interventions, the content of the interventions will differ among participants to some degree due to individual patient needs and the course of the health condition.

### Outcomes

The surveys will take place at baseline (t_0_), after 6 months (t_1_), and after 12 months (t_2_). The primary patient-reported outcome of the study is the mental dimension of the health-related quality of life items (mental component summary [MCS] score; 12 items) from the 36-Item Short Form Health Survey (SF-36), which will be measured as changes in MCS scores from t_0_ to t_2_. We will use the self-report SF-36 questionnaire, which includes retrospective questions about the previous 4-week period. The SF-36 includes 36 questions regarding subjective health status. It has been psychometrically tested and normed for Germany [22,23]. For secondary patient-reported outcomes, the following instruments will be used: (1) the physical dimension of the health-related quality of life items (physical component summary score; 12 items) from the SF-36 [22,23]; (2) the summary score of depression scale from the Patient Health Questionnaire-9 [24]; (3) the short form of the German version of the European Health Literacy Survey Questionnaire (HLS-EU-Q47; HLS-EU-Q16-GER [includes 16 items from the HLS-EU-Q47]) [25]; and 6 scales from the HLS-EU-Q47 [26], Index for the Assessment of Health Impairments [27], and Essen Coping Questionnaire [28].

### Participant Timeline

Multimedia Appendix 1 shows the timeline of study phases, as recommended by the SPIRIT guidelines, for the enrollment, intervention, and assessment time points.

### Sample Size

A power analysis was done a priori. The sample size was calculated with the Power Analysis & Sample Size 16.0.3 program (NCSS LLC) to demonstrate group differences in the primary outcome (MCS scores). The assumptions were based on a study by Markle-Reid et al [20]. The sample size was estimated based on an effect size of 0.50 with a significance level (α) of .05, using a 2-sided (2-tailed), 2-sample unequal variance *t* test (experimental group sample size: mean 55.3, SD 7.8; control group sample size: 53.5, SD 9.6). The maximum number of cases was limited to 130 in order to guarantee that the interventions will be offered to every participant in the intervention group during the limited study period. This maximum sample size of 130 (65 per group), which was calculated based on the aforementioned input parameters, yields 64.1% power to reject the null hypothesis.

### Recruitment

Recruitment was conducted for 5 months, from August (the first patient was recruited on August 10, 2021) to the end of December 2021, in cooperation with the multiprofessional team (physicians, physician assistants, clinical psychologists, social workers, and midwives) at the CHC. The eligibility of participants was determined by the CHC staff, who were trained in advance. Based on the inclusion criteria, the staff identified patients with at least 1 chronic diagnosis and then contacted them directly. A recruitment protocol was used. Patients received a letter of information that included notes on the study aims, study design, data protection, and randomization. The information was available in 6 languages—Turkish, Macedonian, Albanian, English, French, and German. Participation is voluntary, and consent can be withdrawn at any time without incurring disadvantages in treatment. In addition, once per week, a nurse (and a bachelor of arts student) supported the recruitment process at the CHC in order to clarify questions. After verbal consent was obtained, patients were contacted by a research assistant by telephone for further information. Questionnaires were designed as self-report surveys and were administered after a 48-hour consideration period and the provision of written consent. If there is a need for assistance, home interviews or interviews at the CHC will be offered by study assistants.

### Assignment of Interventions (for Controlled Trials)

#### Sequence Generation

After the baseline survey, a statistician from the Institute of Medical Biometry and Epidemiology at the Medical Center Hamburg-Eppendorf randomly assigned included study participants to the intervention group or control group (stratified by gender). Randomization was realized via a computer-based permuted block design with variable block lengths to generate the allocation sequence, in which the number of assignments to the intervention group satisfied the 1:1 allocation ratio. The allocation concealment mechanism is described in the next subsection.

#### Data Collection, Management, and Analysis

After randomization, the interventionists were informed about the group assignments because they are the ones who will implement the interventions. This was also necessary for informing the participants about the allocations. The research assistant who is responsible for data extraction and analyses was blinded and excluded from the entire recruitment process.

#### Data Collection Methods

Data will be collected primarily at baseline (t_0_), after 6 months (t_1_), and after 12 months (t_2_). At baseline, data will be collected on the primary and secondary outcomes (described in the *Outcomes* section), in addition to variables on diseases, demographics, socioeconomic factors, the utilization of health services, and digital mobile health apps. The flow of participants through the study phases has been presented in a flow diagram that conforms to the CONSORT (Consolidated Standards of Reporting Trials) guidelines [29] for pragmatic trial reporting (Multimedia Appendix 2).

#### Data Management

Surveys will be realized as paper and pencil assessments. For data input, descriptive statistics and statistical analyses, which will be conducted with SPSS Statistics version 22 (IBM Corporation), will be used. The double entry of data (primary data) has been planned for the first 10 questionnaires, and random checks will be performed as data entry progresses to check for input errors. Collected data from the baseline and follow-up assessments will be checked for missing data, plausibility, and outliers.

### Statistical Analysis

For analyses, data from the intention-to-treat population will be used. Data from the baseline survey will be analyzed descriptively to compare the demographic, socioeconomic, and clinical characteristics of study participants. Means and SDs or medians and IQRs will be calculated for continuous variables, and absolute frequencies and proportions will be calculated for categorical variables. For the primary analysis, a mixed linear regression model will be used, with groups, time points, and gender included as fixed effects; participants included as random effects; and baseline values included as covariates. For the primary analysis, the treatment effect at t_2_ will be evaluated, and the 2-sided significance level (α) will be set to 5%. To summarize the characteristics and outcomes of each group, as well as for the comparison of the groups, descriptive statistics and appropriate tests will be used. For regression models, gender will be included as a fixed effect, as per the stratification used in the randomization process. Other covariates (eg, age and the number and duration of diagnoses of chronic illness) will be included in sensitivity analyses. Following the study analysis procedures of Markle-Reid et al [20], the correlation between the quantity of utilized interventions and the primary outcome will be analyzed.

### Ethics Approval

Ethics approval was obtained from the institutional Competence Health Center’s ethics commission at the University of Applied Sciences Hamburg in March 2021 (procedure number: 2020-14). The study will be conducted in accordance with the commission’s ethical standards and the Helsinki Declaration.

## Results

The RCT was developed to evaluate a community health nurse model for primary care settings. Funding for conducting the CoSta study was provided by the Federal Ministry of Education and Research Germany in 2020 (contract number: 13FH019SX8). In total, 187 participants were recruited at the beginning of August 2021. Further, 92 were excluded and 94 were randomized. Surveys will be conducted until the end of 2022. A summary of the results will be published in peer-reviewed journals and presented at scientific conferences after the completion of the t_2_ surveys (ie, by the end of 2022).

## Discussion

### Study Overview

The CoSta study will evaluate a community health nurse–led intervention for enhancing the well-being of patients with chronic health conditions in an urban neighborhood. By using a randomized trial design, the feasibility and benefits of the CoSta intervention will be compared to those of usual treatment. Thus, the study will focus on advanced nursing practitioners’ role in Germany and will offer an approach to targeting the needs-oriented care of patients with chronic illnesses.

Via a randomized group comparison, the analyses will generate data on health-related quality of life, depressive symptoms, anxiety, health literacy, coping, and the utilization of health care in the study population. Furthermore, influencing social aspects and local contexts will be taken into consideration.

The study results could contribute to informing stakeholders in the health care system about responsibilities that can be applied universally in primary care. The expected results could reduce the research-to-practice gap, and they could serve as a basis for the transfer the care model to other regions and the German health care system.

### Strengths and Limitations

A strength of the CoSta study and the nurse-led model is the integration of a multiprofessional team within the CHC. Additionally, the study design can be applied directly in the field. The study will be conducted with the cooperation of the study participants. The CoSta study will provide evidence-based information about a new approach in health care for providing a better quality of life to patients with (multiple) chronical illnesses in a more socially disadvantaged neighborhood. The study results can be used to support other ongoing projects [13,14] that target the anchoring of CHN in Germany.

Due to the field conditions however, there are limitations that can be expected throughout the study period. First, there will probably be a greater need for trained staff during the entire study period. Particularly for the recruitment and education of study participants before study enrollment, the study nurse was a supportive team member for recognizing barriers at an early stage. Second, due to the global COVID-19 pandemic, it was difficult to identify and recruit patients with chronic illnesses. As such, a longer period for recruitment would have been useful.

### Harms

The participants are a vulnerable group due to their chronic health conditions. As experienced nurses, the researchers involved will be able to engage appropriately with the patients, manage spontaneous adverse events, and terminate participation (if necessary). For participants, there will be opportunities to ask questions before and during the data collection process and trial interventions.

## Appendix

Multimedia Appendix 1 Schematic diagram of the Community Health Nursing in der Stadt study phases, as recommended by the SPIRIT (Standard Protocol Items: Recommendations for Interventional Trials) guidelines.

Multimedia Appendix 2 Flow diagram of progress from enrollment to analysis (CONSORT [Consolidated Standards of Reporting Trials] 2010 flow diagram).

## Abbreviations

CHC: community health center
CHN: community health nursing
CONSORT: Consolidated Standards of Reporting Trials
CoSta: Community Health Nursing in der Stadt
HLS-EU-Q16-GER: short form of the German version of the European Health Literacy Survey Questionnaire
HLS-EU-Q47: European Health Literacy Survey Questionnaire
MCS: mental component summary
RCT: randomized controlled trial
SF-36: 36-Item Short Form Health Survey
SPIRIT: Standard Protocol Items: Recommendations for Interventional Trials

## Data Availability

The data sets that will be generated during the study will be available from the corresponding author on reasonable request.
