# Supplementary figures and images for: A Care Concept of Community Health Nursing Interventions for Adults With Chronic Health Conditions in an Urban Area: Protocol for a Randomized Controlled Field Trial (CoSta Study)
Source: JMIR Res Protoc. 2022 Sep 28;11(9):e37965. doi: 10.2196/37965 (PMC9557981; doi:10.2196/37965)

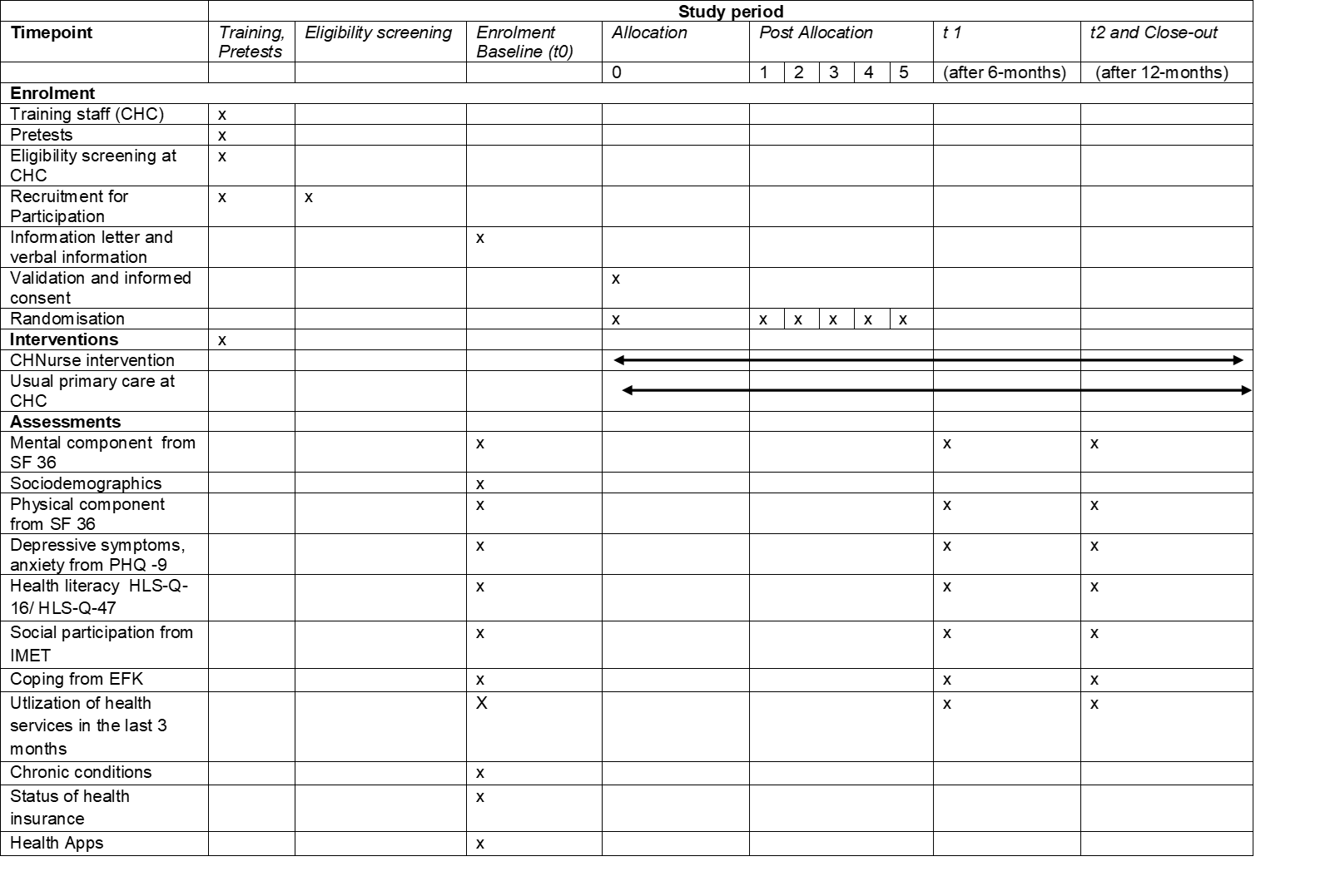

Supplement: Multimedia Appendix 1 [file resprot_v11i9e37965_app1.png]

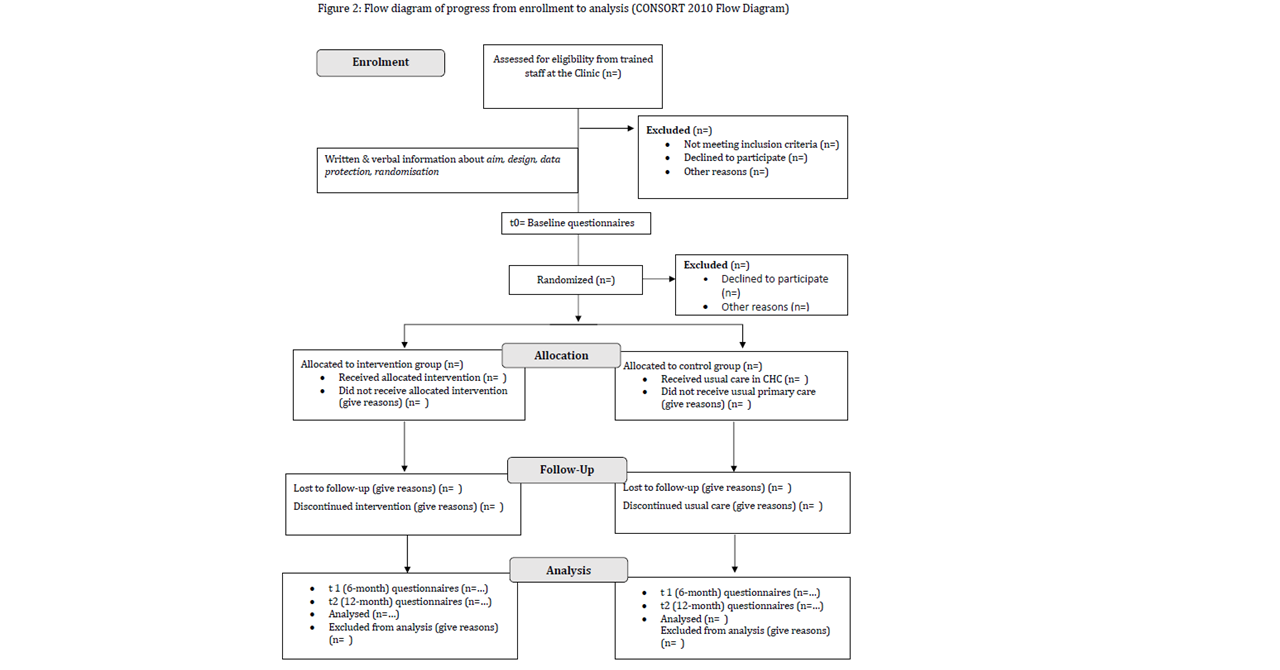

Supplement: Multimedia Appendix 2 [file resprot_v11i9e37965_app2.png]
